# Ringer’s Lactate Hydration and Incidence of Post ERCP Pancreatitis: A Descriptive Cross-sectional Study

**DOI:** 10.31729/jnma.5435

**Published:** 2020-09-30

**Authors:** Ashis Pun, Amit Dhungana, Dipendra Neupane

**Affiliations:** 1 Department of Surgery, Bharatpur Hospital, Bharatpur, Chitwan, Nepal; 2 Department of Anaesthesiology, Bharatpur Hospital, Bharatpur, Chitwan, Nepal

**Keywords:** *endoscopic retrograde cholangiopancreatography*, *pancreatitis*, *ringer’s lactate*

## Abstract

**Introduction:**

Endoscopic retrograde cholangiopancreatography is one of the most frequently used treatment modality for various pancreatobiliary problems. Frequent complications of endoscopic retrograde cholangiopancreatography include pancreatitis, cholangitis, hemorrhage and perforation. This study was done to see the prevalence of post endoscopic retrograde cholangiopancreatography pancreatitis in patient aggressively hydrated with Ringer's Lactate solution.

**Methods:**

A descriptive cross sectional study was carried out on patient undergoing endoscopic retrograde cholangiopancreatography at Bharatpur Hospital from June 2018 to August 2020. Ethical clearance was taken from Institutional Review Committee Bharatpur Hospital (reference number 16/076/77). The convenient sampling method was applied. Data were collected and analyzed in statistical package for the social sciences version 16. Point estimate at 95% confidence interval was calculated along with frequency and proportion for binary data.

**Results:**

Pain abdomen was assessed using Visual Analogue Scale and it was found that 8.1% of patients (15 patients) complained of pain abdomen with visual analogue scale > 3. Serum amylase was sent only in those patients who complained of pain abdomen and only in three patients (1.6%) serum amylase was increased more than 3 times the upper limit of normal value suggestive of pancreatitis. All three patients who had pancreatitis had precut sphincterotomy.

**Conclusions:**

In this study we found that incidence of pancreatitis slumped after aggressive hydration with Ringer's lactate solution and adjunct use of other prophylactic measures for prevention of post endoscopic retrograde cholangiopancreatography pancreatitis might yield further better results.

## INTRODUCTION

Post Endoscopic Retrograde Cholangiopancreatography Pancreatitis (PEP) is the most common complication of Endoscopic Retrograde Cholangiopancreatography (ERCP). It ranges from 2%-10% and upto 40% in high risk cases.^1^ Various procedural techniques and pharmacological interventions have been investigated.

Aggressive hydration with Ringer's lactate (RL) fluid has been found to have promising result. Anti-inflammatory effect of RL has two possible explanations. First, lactate in RL gets metabolized to bicarbonate in liver which results in lowering metabolic acidosis whereas, normal saline (NS) when given in large volumes produces dilutional hyperchloremic acidosis due to high sodium and chloride content. Besides, plasma bicarbonate concentration decreases as chloride concentration increases. Studies show acidosis enhances inflammation and necrosis in acute pancreatitis. Second, RL may directly decrease inflammatory response in these patients by preventing activation of nuclear factor kappa B (NF-KB) transcription factor involved in the inflammatory process.^2,3^

Therefore, this study was carried out to find out the incidence of PEP after aggressive hydration with RL in patients undergoing ERCP.

## METHODS

A descriptive cross sectional study was conducted in the Department of Surgery, Bharatpur Hospital from June 2018 to August 2020. All the procedures were performed by same endoscopic surgeon in the same operative setting. Ethical approval was taken from the institutional review committee (IRC) Bharatpur Hospital (reference number 16/076/77).

The sample size was calculated using the following formula,

Sample size (n)= z^2^p(1-p)/(e)^2^, where

z = 1.96 at 95% CI
p (prevalence)= 50%
e (precision)= 5%
Sample size (SS) = 384

Now using Prevalence formula for finite population, No of patients undergoing ERCP in last 15 months = 300 approx.

Thus corrected sample size will be

$$\text{1}+\frac{\text{n}}{\frac{\text{n}-\text{1}}{\text{Population}}}$$

Where, n = 384

Population = 300

Corrected Sample size becomes 169 and adding 10 % drop outs, sample size is estimated to be 186.

Patients were aggressively hydrated with ringer lactate solution (3 ml/kg/hr) during procedure, 20 ml/kg bolus immediately after procedure, and 3 ml/kg/hr for 8 hours post procedure.^4,5^ Patient at risk of fluid overload were excluded from the study. Serum amylase >3 times the upper limit of normal was defined as PEP. Serum amylase was only sent if patient complain of pain (visual analogue score>3) persisting more than 8 hours.^6^ Injection diclofenac 75mg intramuscular was given during procedure. Only guidewire cannulation technique was used. Five French (Fr.) pancreatic duct (PD) stent was placed if PD cannulated >3 times.

Inclusion criteria: All patients undergoing ERCP with American Society of Anesthesiologist Physical Status (ASA-PS) I and II, with at least one risk factor for pancreatitis which may be procedure related or patient factor were included.

Exclusion criteria:

- Acute pancreatitis
- Congestive heart failure
- Respiratory insufficiency
- Severe liver disease
- Hypo or hypernatremia

Data analysis was done in the statistical package for the social sciences (SPSS) version 16. Point estimate at 95% confidence interval was calculated along with frequency and proportion for binary data.

## RESULTS

A total of 186 patients undergoing ERCP were enrolled in this study. Each patient had a minimum risk factor for pancreatitis. During the post operative period, pain abdomen was assessed using Visual analogue Scale (VAS) and it was found that 15 patients (8.1%, C.I 95% patients) complained of pain abdomen with VAS>3. Serum amylase was sent only in those patients who complained of pain abdomen and only in three patients (1.6%, C.I 95%) serum amylase was increased more than 3 times the upper limit of normal value suggestive of pancreatitis.

Of these 186 patients, 55 patients (29.6%) were male and 131 patients (70.4%) were female. Similarly, 23 patients (12.4%) were American Society of Anaesthesiologist-physical status (ASA-PS) I and 163 patients (87.6%) were ASA-PS II.

During the procedure, ease of cannulation into ampulla of vater was assessed and it was found that in 123 patients (66.1%), cannulation was easy. However, 63 patients (33.9%) had difficult cannulation. Similarly, precut sphincterotomy, pancreatic duct cannulation, and continuous radial expansion were performed in these patients as shown (Table 1).

**Table 1:** Procedure Performed.

Procedure	Performed n (%)	Not performed n (%)
Precut Sphincterotomy	46 (24.7%)	140 (75.3%)
Pancreatic Duct Cannulation	18 (9.7%)	168 (90.3%)
Continuous radial expansion	29 (15.6%)	157 (84.4%)

Mean age of patients who had pain abdomen was 61±15.847 years and mean age of patient who did not have pain abdomen was 51.09±15.938 years. However, mean age of patients with serum amylase raised more than 3 times the upper limit of normal was 38.67±17.786 years.

**Figure 1:**
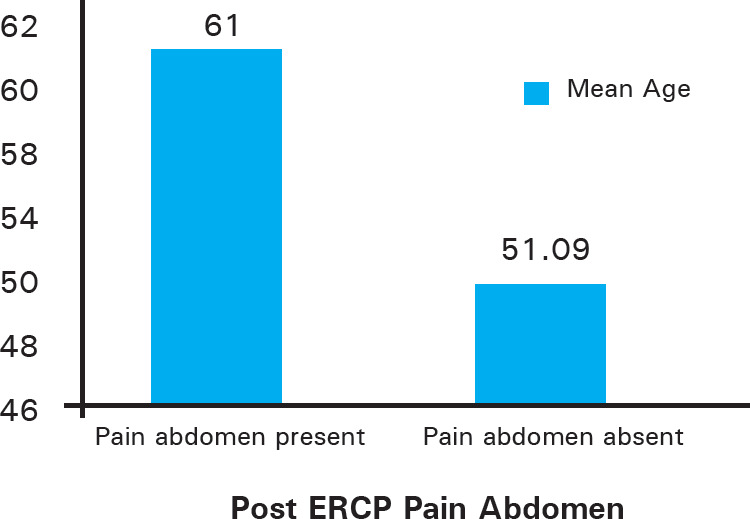
Post ERCP Pain Abdomen.

Similarly, post ERCP pain abdomen was significantly higher in patient with difficult cannulation compared to easy cannulation. Thirteen patients with difficult cannulation had pain abdomen compared to two patients with easy cannulation. All three patients who had pancreatitis (pain abdomen with increased serum amylase > 3 times) had difficult cannulation while none patient with easy cannulation had pancreatitis.

**Figure 2:**
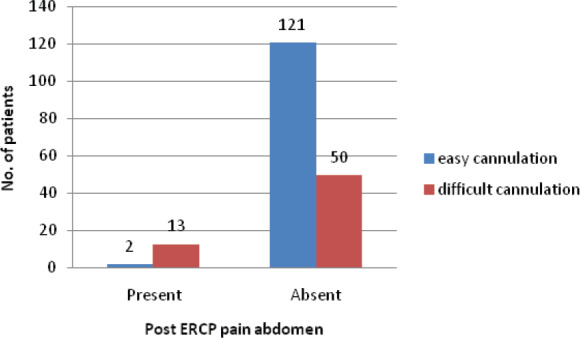
Ease of cannulation and post ERCP pain Abdomen

Incidence of post ERCP pain abdomen in patients who had precut sphincterotomy, pancreatic duct cannulation and continuous radial expansion was as follows (Table 2).

**Table 2:** Post ERCP pain abdomen.

		Pain abdomen Present	Pain Abdomen Absent
Precut sphincterotomy	Performed	11	35
	Not performed	4	136
Pancreatic Duct cannulation	Performed	0	18
	Not Performed	15	153
Continuous radial expansion	Performed	2	27
	Not Performed	13	144

Similarly, all three patients who had pancreatitis had precut sphincterotomy. None of the patient who had undergone pancreatic duct stenting and continuous radial expansion developed pancreatitis.

## DISCUSSION

Pancreatitis is the most frequent complication following ERCP attributed upto 40% in high risk cases. Various methods and techniques are studied and implicated for the prevention of pancreatitis. Guidewire technique of common bile duct (CBD) cannulation significantly lowered the incidence of PEP compared with the contrast-assisted method (RR 0.51; 95 % CI, 0.32-0.82).^4,5^. Recent meta-analyses calculated an odds ratio of 0.44 for rectal non-steroidal anti-inflammatory drugs (NSAIDs) and 0.35 for pancreatic duct (PD) stents.^7–10^

However, controversy exists on how PEP should be defined. Most commonly Cotton, et.al and revised Atlanta consensus of acute pancreatitis are widely accepted. Freeman et al reported that the risk factors for post-ERCP pancreatitis were both patient-related factors (i.e. female sex, young age, suspected sphincter of oddi dysfunction) and procedure-related factors (i.e. difficult cannulation, pancreatic duct injection, and precut sphincterotomy).^11^ In their study, the multivariate analyses of independent risk factors for post-ERCP pancreatitis demonstrated statistical association with cannulation difficulty (OR, 3.9; 95% CI, 1.7-8.6), dilation of an intact biliary sphincter (OR, 3.9; 95% CI, 1.5-9.9), and non-use of vigorous hydration (OR, 2.4; 95% CI, 1.1-5.2).^11^

One of the methods for reducing incidence of pancreatitis is early vigorous hydration. The rationale behind vigorous hydration is to resolve hypovolemia so that perfusion is adequate. But now choice of fluid is shifted toward Ringer's lactate solution because of more suitable acid base balance and its anti-inflammatory property. However, there is still no definite evidence towards use of RL but results are encouraging in favour of RL.^2,12–14^ Therefore, American Society of Gastroenterology (ASGE) and European Society of Gastroenterology (ESGE) also supports RL hydration in medically fit patients.

In a 62 patient pilot study, aggressive hydration with ringer lactate solution demonstrated significant reduction in PEP compared to standard hydration (0% vs 17%; P = 0.016). Similarly, in a randomized, double-blinded, controlled study of 150 patients PEP was noted in 5.3% of patients receiving aggressive hydration compared with 22.7% receiving standard hydration (P = .002).^4^ In a systemic review and meta-analysis done by Wu D and collegues on an efficacy of aggressive hydration with Ringer's Lactate published in 2017 showed lower incidence of pancreatitis after ERCP (OR = 0.29; 95% confidence interval (CI), 0.16-0.53); a lower incidence of moderate to severe PEP (OR = 0.16; 95% CI, 0.03-0.96); lower incidence of hyperamylasemia (OR = 0.38; 95% CI, 0.25-0.59); lower risk of pain (OR = 0.17; 95% CI, 0.08-0.38); and a shorter duration of hospital stay (standardized mean difference = -0.41; 95% CI, -0.69 to -0.14).^15^ Moreover, Zhang ZF, et al performed meta-analysis of randomized trial on aggressive Ringer's Lactate (RL) also found RL as an effective and safe therapy for prophylaxis of PEP.^16^

Park, et.al studied role of aggressive fluid therapy in three groups with aggressive RL, aggressive NS and normal hydration with RL. His study also showed significant difference in the PEP rate between the aggressive RL group (3.0 %, 95 % confidence interval [CI] 0.1 % - 5.9 %; 4 /132), the aggressive NS group (6.7 %, 95 %CI 2.5 % - 10.9 %; 9 /1 34) and the standard RL group (11.6 %, 95 % CI 6.1 % - 17.2 %; 15 /129; P = 0.03).^17^ In our study also marked reduction in incidence of PEP was seen (1.6% on aggressive Ringer's Lactate hydration protocol.

Besides for reduction of PEP rectal administration of NSAIDS is found to be effective in various study.^18,19,20^ Mok SRS et al. randomized, double blinded, placebo-controlled trial showed reduced incidence of PEP in ringer lactate plus rectal indomethacin versus normal saline plus placebo.^21^ In our study we have also included NSAIDs (diclofenac intramuscular) in all cases however, various studies support effectiveness of NSAIDs given only per rectal compared to other routes. But in our country rectal NSAIDS is not easily available so we don't practice giving prophylactic rectal NSAIDs for pancreatitis. In 2014, Ignasi Puigetal performed meta-analysis and also showed supporting evidence with rectal diclofenac or indomethacin given either before or after procedure but, there was no evidence to support oral or parenteral administration.^22–24^

## CONCLUSIONS

Our study demonstrates that the introduction of selective and routine use of aggressive hydration with Ringer's lactate significantly reduces the incidence of pancreatitis. However, there are some confounding factors in our study like we had used guidewire cannulation technique, PD stents if PD cannulation >3 times and intramuscular diclofenac which might have also influenced in our result. Therefore, we conclude that aggressive hydration with RL reduces PEP and combining RL hydration with other methods for decreasing PEP may offer even better results.

## Conflict of Interest

**None.**
